# Re-Audit of Safe Deprescribing on Inpatient Psychiatric Wards After Implementation of an Electronic Prescribing Management and Administration (EPMA) System in an NHS Trust

**DOI:** 10.1192/bjo.2025.10536

**Published:** 2025-06-20

**Authors:** Udoka Onyechere, Toibat Adeyinka, Harvir Sahota

**Affiliations:** 1 Cumbria, Northumberland, Tyne and Wear Foundation NHS Trust, Newcastle, United Kingdom; 2 Coventry and Warwickshire Partnership NHS Trust, Coventry, United Kingdom

## Abstract

**Aims:** The audit aimed to evaluate the effectiveness of transitioning from paper-based patient prescription charts (Kardex) to an electronic prescribing and medication administration system (EPMA) in improving compliance with safe deprescribing practices on inpatient psychiatric wards. Specific objectives included assessing adherence to Trust guidelines, reducing incidents of incorrect medication deprescription, and enhancing clarity regarding medication changes.

**Methods:** This audit was performed in May 2024 on all psychiatric inpatient wards utilising the EPMA system. This system had been in use for over a year in the Trust following a phasing out of the paper Kardex. During this period, the EPMA records of inpatients were evaluated. The findings were compared with that from a previous audit, which examined Kardex records in March 2022. The comparative analysis centred on deprescribing practices, examining whether medications were properly discontinued, entries were completely filled, and justifications for deprescribing were noted. The audit complied with Trust protocols and ethical governance requirements.

**Results:** The transition from the Kardex system to EPMA resulted in significant improvements in safe deprescribing practices. There was 100% compliance in details on the system corresponding to most of the standards measured in the previous audit, including name crossed, row crossed fully, ID, code (reason) and stop date. The sole exception to this was observed when utilising the 'other’ option in EPMA’s dropdown menu, where adherence to providing a stated reason was 94.5%, a metric not evaluated in the initial audit as this was not facilitated by the paper Kardex. In this audit, all the standards were met and the medications were considered safely deprescribed. This stands in contrast to the previous audit where less than 33.88% of deprescribed medications met the standards.

**Conclusion:** The EPMA system demonstrated substantial progress in promoting safe deprescribing practices aligned with Trust guidelines. The notable improvement in compliance clearly demonstrates the significant influence of technology on clinical practice and patient safety in relation to medication prescription and administration in this case.

